# Sated by a Zero-Calorie Sweetener: Wastewater Bacteria Can Feed on Acesulfame

**DOI:** 10.3389/fmicb.2019.02606

**Published:** 2019-11-20

**Authors:** Sabine Kleinsteuber, Thore Rohwerder, Ute Lohse, Bettina Seiwert, Thorsten Reemtsma

**Affiliations:** ^1^Department of Environmental Microbiology, Helmholtz Centre for Environmental Research – UFZ, Leipzig, Germany; ^2^Department of Analytical Chemistry, Helmholtz Centre for Environmental Research – UFZ, Leipzig, Germany; ^3^Institute for Analytical Chemistry, University of Leipzig, Leipzig, Germany

**Keywords:** micropollutants, trace organic compounds, biotransformation, biodegradation, amidohydrolase, sulfohydrolase, microbial adaptation, organic contaminant

## Abstract

The widely used artificial sweetener acesulfame K has long been considered recalcitrant in biological wastewater treatment. Due to its persistence and mobility in the aquatic environment, acesulfame has been used as marker substance for wastewater input in surface water and groundwater. However, recent studies indicated that the potential to remove this xenobiotic compound is emerging in wastewater treatment plants worldwide, leading to decreasing mass loads in receiving waters despite unchanged human consumption patterns. Here we show evidence that acesulfame can be mineralized in a catabolic process and used as sole carbon source by bacterial pure strains isolated from activated sludge and identified as *Bosea* sp. and *Chelatococcus* sp. The strains mineralize 1 g/L acesulfame K within 8–9 days. We discuss the potential degradation pathway and how this novel catabolic trait confirms the “principle of microbial infallibility.” Once the enzymes involved in acesulfame degradation and their genes are identified, it will be possible to survey diverse environments and trace back the evolutionary origin as well as the mechanisms of global distribution and establishment of such a new catabolic trait.

## Introduction

The potassium salt of acesulfame, named acesulfame K (ACE-K; CAS = 55589-62-3; E950 in the EFSA list of food additives) is an artificial sweetener that was discovered in 1967 and first described by Clauß and Jensen (1973). Since its first approval in the UK in 1983, ACE-K has been increasingly used as zero-calorie sweetener in beverages, food, personal care products, and pharmaceuticals in more than 100 countries worldwide (Klug and von Rymon Lipinski, 2012; Lange et al., 2012). It is 200 times sweeter than sucrose and stable under heat as well as moderately acidic or alkaline conditions (Klug and von Rymon Lipinski, 2012), making it suitable as additive for baking and products that require a long shelf life. After ingestion, the acesulfame anion (ACE) is readily absorbed but not metabolized in the human body and eventually excreted unchanged *via* the kidneys (Klug and von Rymon Lipinski, 2012), thus ending up in domestic wastewater. An EU-wide survey detected ACE in 93% of the effluents from 90 wastewater treatment plants (WWTPs) in 18 European countries (Loos et al., 2013). Due to its limited removal in municipal WWTPs (Harwood, 2014; Tran et al., 2015) and its hydrophilicity and mobility in aquatic environments, ACE has also been used as chemical marker for domestic wastewater input into surface waters and groundwater (Buerge et al., 2009; Scheurer et al., 2011; Robertson et al., 2016). However, recent studies have disproved the recalcitrance of ACE in WWTPs (Castronovo et al., 2017; Kahl et al., 2018), leading to the hypothesis that this previously persistent, xenobiotic compound has become biodegradable by microbial adaptation to a novel carbon source.

## Ubiquitous Distribution of Acesulfame in Aquatic Environments

Due to its worldwide use and persistence in WWTPs, ACE has been detected in all environmental compartments impacted by anthropogenic discharges. The ubiquitous occurrence of ACE in aquatic environments was first reported by Buerge et al. (2009) and Scheurer et al. (2009), who found ACE concentrations in the range of 1–10 μg/L in surface waters, groundwater, and even tap water. Buerge et al. (2009), for example, detected ACE consistently in effluents of 10 WWTPs in almost equal concentrations as in the influents, and in 65 out of 100 investigated groundwater wells. Underpinning its recalcitrance, Scheurer et al. (2009) found ACE being the most persistent trace compound when comparing the efficiency of WWTP and soil aquifer treatment in removing seven commonly used artificial sweeteners. In line with this, ACE concentrations in eight Swiss lakes were shown to correlate with the anthropogenic burden by domestic wastewater, i.e., the ratio of population in the catchment area and mean water through-flow (Buerge et al., 2009).

Similar results were obtained with water samples collected between 2007 and 2009 (Spoelstra et al., 2013) as well as in 2012 and 2013 (Liu et al., 2014) from the Grand River (Ontario, Canada), showing that ACE persisted over 300 km in the river, which reflects the cumulative human population in the watershed and the effluents from 30 WWTPs. ACE was also detected in the groundwater of other urban areas in Canada (Van Stempvoort et al., 2011) as well as in a 15-year-old septic system plume in Ontario (Robertson et al., 2013). Also in 2013, the widespread distribution of ACE in surface waters and groundwater in Germany was proven (Nödler et al., 2013). ACE detection frequencies were 100% in groundwater samples (median concentration 14 ng/L), 96% in surface water samples (median concentration 1.219 μg/L), and 100% in finished tap water, with concentrations of 1.98–2.44 μg/L in Berlin tap water.

More recently, Schaider et al. (2016) detected ACE in 17 out of 20 domestic drinking water wells in Massachusetts (USA) tested for organic wastewater compounds. In addition, several other sampling campaigns between 2011 and 2014 proved the ubiquitous distribution and persistence of ACE in aquatic environments in the area of Tianjin, China (Gan et al., 2013), in rivers and lakes in Finland (Perkola and Sainio, 2014), in the Esmeraldas watershed in Ecuador (Voloshenko-Rossin et al., 2015), in coastal waters of the Mediterranean Sea (Nödler et al., 2016), and in small rivers in Germany and Sweden (Li et al., 2016).

## Emerging Biodegradability of Acesulfame

In the last years, there has been increasing evidence that besides abiotic processes such as photolysis, also biodegradation contributes to ACE attenuation under suitable conditions. In soil incubation experiments, spiked ACE was degraded with half-lives of 3–49 days depending on the soil type, while no degradation was observed in sterilized soil (Buerge et al., 2011). Other experiments indicated that ACE degradation is temperature-dependent, as ACE was degraded in soil columns and in aquifer sediment at 20°C but not at 6°C (Burke et al., 2014, 2017). ACE was not degraded under nitrate-reducing, iron−/manganese-reducing, or sulfate-reducing conditions (Burke et al., 2017). These clear hints on biodegradation are in contrast to the aforementioned studies showing that ACE is persistent in WWTPs. Buerge et al. (2011) explained this contradiction by different microbial communities in soils and activated sludge or different conditions and short retention times in WWTPs. Tran et al. (2014) suggested co-metabolic ACE degradation by autotrophic ammonia-oxidizing bacteria, while Gan et al. (2014) discussed a combined effect of photolysis and biodegradation contributing to ACE transformation under environmental conditions. Taken together, these studies have shown that ACE might be slowly degraded in soils and aquifer sediments but substantial removal in WWTPs was not evident until 2016. In a study by Storck et al. (2016), biodegradation tests in a lab-scale fixed-bed reactor revealed that ACE (initial concentration ≈10 μg/L) was completely degraded in WWTP effluents within 17 days after a lag phase of 11 days. Falas et al. (2016) reported ACE removal of up to 80% in bench-scale reactors with activated sludge from municipal wastewater. Cardenas et al. (2016) measured ACE removal of >90% in a WWTP in Queensland (Australia).

Evidence of ACE biodegradation in conventional biological wastewater treatment was shown by Castronovo et al. (2017), who detected removal between 59 and 97% in 13 municipal WWTPs and identified sulfamic acid (SA) as the mineralization product. ACE degradation under aerobic as well as under nitrate-reducing conditions was confirmed in bench-scale nitrifying/denitrifying sequencing batch reactors (SBRs) as well as in aerobic batch experiments with activated sludge and sand filters, with stoichiometric formation of SA. No relation of ACE removal with nitrification rate or ammonium concentration as suggested by Tran et al. (2014) was observed. Kahl et al. (2018) observed a clear seasonality of ACE removal in a WWTP and an aerated treatment wetland over an entire year. They reported ACE degradation of >85% during summer and autumn in nine German WWTPs, with annual removal performance being more stable in larger plants, enhanced by low biological oxygen demand and impeded by water temperatures below 10°C. The ongoing establishment of the biodegradation potential in WWTPs is illustrated by the decreasing ACE content in surface waters: between 2013 and 2016, the ACE mass load in the German rivers Elbe and Mulde decreased by 70–80% (Kahl et al., 2018).

## Microbial Key Players in Acesulfame Catabolism

In our previous study, complete catabolic degradation of ACE as sole carbon source was demonstrated for the first time by enrichment of ACE-degrading bacteria in sludge-free mineral medium (Kahl et al., 2018). Only the carbonaceous fraction of ACE was completely removed while SA was formed as mineralization product. Microbial community analysis by 16S rRNA amplicon sequencing revealed enrichment of several alphaproteobacterial genera (e.g., *Bosea*) during prolonged cultivation on ACE; however, we failed to isolate ACE-degrading pure cultures (Kahl et al., 2018).

**Figure 1:**
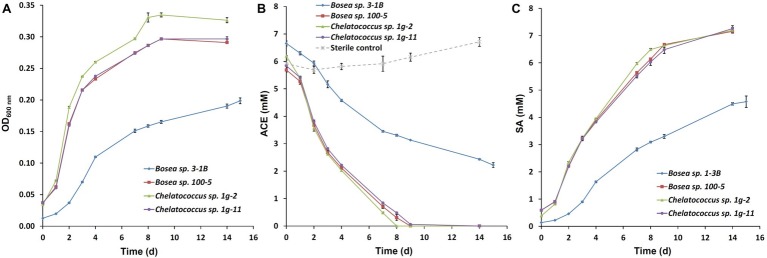
Growth curves and time course of acesulfame anion (ACE) degradation of the four bacterial strains *Bosea* sp. 3-1B, *Bosea* sp. 100–5, *Chelatococcus* sp. 1-2 g, and *Chelatococcus* sp. 1g-11 on 1 g/L acesulfame K in Brunner medium (DSM 462) at 30°C and 150 rpm. **(A)** Optical density (OD) at 600 nm; **(B)** degradation of ACE; **(C)** formation of sulfamic acid (SA). Batch cultures were set up in triplicates and inoculated 1:10 from pre-cultures grown in the same medium. Additionally, one sterile control per strain was set up. ACE and SA concentrations in supernatants were measured by ion chromatography as described by Kahl et al. (2018). Data points are mean values (*n* = 3 for the cultures, *n* = 4 for the sterile controls), error bars indicate the standard deviation. Note that the cultures contained remaining SA from the pre-cultures from the beginning. In the sterile controls, SA concentrations were below the detection limit (<0.5 mg/L).

Recently, we succeeded to isolate slow-growing ACE degraders from the enrichment cultures B and b (Figure 4 in Kahl et al., 2018), which form small colonies on R2A agar and were all identified as *Bosea* sp. (strain 3-1B, acc. no. MN270877). Further enrichments inoculated directly with sludge samples from the treatment wetland (100 mg/L or 1 g/L ACE-K as sole carbon source in Brunner medium DSM 462, 30°C) resulted in the isolation of several other strains identified as *Bosea* sp. (strain 100–5, acc. no. MN270878; isolated on 100 mg/L ACE-K) and *Chelatococcus* sp. (strain 1g-2, acc. no. MN270879; strain 1g-11, acc. no. MN270880; both isolated on 1 g/L ACE-K). Growth curves and ACE degradation in aerobic batch cultures of the four strains are illustrated in Figure 1. Three of the strains degraded 1 g/L ACE-K within 8–9 days (Figure 1B) while forming stoichiometric amounts of SA (Figure 1C). The strains prefer organic acids as growth substrates (succinate, pyruvate, acetate, and 3-hydroxybutyrate were tested) but grow poorly on sugars (glucose, fructose). Interestingly, an ACE-degrading *Chelatococcus* sp. strain was recently isolated from activated sludge of a WWTP in China (Huang et al., 2019). The authors identified their strain YT9 as *C. asaccharovorans*, which was also the closest species to our strains 1g-2 and 1g-11.

Cultures of our four isolates incubated with either ACE or 3-hydroxybutyrate as sole carbon source were searched for transformation products of ACE by non-target screening using liquid chromatography-electrospray ionization-high resolution mass spectrometry. In all cultures growing on ACE but not in the corresponding control experiments with 3-hydroxybutyrate, acetoacetamide-N-sulfonic acid (ANSA) was detected in the culture supernatant and identified by its exact mass and fragment ions according to Castronovo et al. (2017).

## Discussion

Previous studies have concluded that ACE is recalcitrant to biodegradation or in the best case transformed to a minor extent by co-metabolic processes, leading to the perception that this micropollutant can be used to trace wastewater input in surface waters. In the light of recent observations, the use of ACE as conservative tracer is obsolete. It has now been demonstrated that bacterial ACE degradation is a catabolic process leading to complete mineralization or assimilation of the carbonaceous part of the molecule, raising questions on the reasons for the long time observed recalcitrance and the biodegradation pathway.

**Figure 2:**
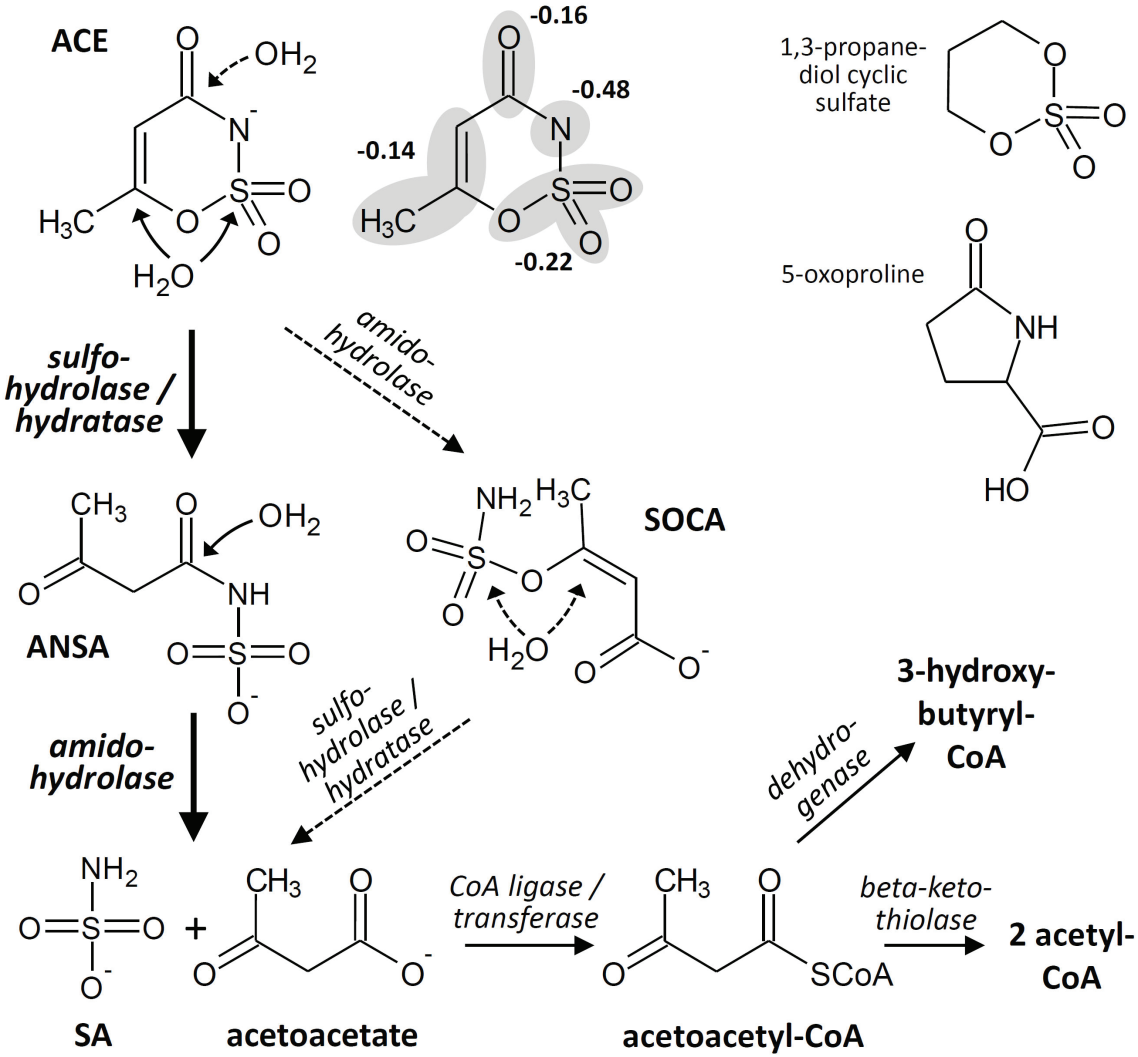
Hypothetical acesulfame anion (ACE) degradation pathway in the isolated bacterial strains and examples for structures of biodegradable cyclic sulfates and amides. The ACE structure is depicted both with the negative charge completely assigned to the amide nitrogen and delocalized over the various molecule fragments according to Popova et al. (2012). ANSA, acetoacetamide-N-sulfonic acid; SA, sulfamic acid; SOCA, 3-(sulfamoyloxy)crotonic acid.

Basically, enzymatic attack of ACE has to cope with the same molecular properties that lead to chemical stability toward hydrolysis and other abiotic decomposition reactions. Formally, ACE combines the properties of (1) a carboxylic acid amide and (2) a sulfamic acid ester in a 6-membered ring structure that is almost planar and resonance-stabilized. While the ring system carries two electron-withdrawing moieties, it is electron-rich owing to the negative charge. Often, the latter is formally assigned to the amide nitrogen but according to quantum chemical calculations the negative charge is delocalized over the molecule (Popova et al., 2012), with 22% at the sulfonyloxy group and only 48% at the amide nitrogen (Figure 2). Consequently, hydrolytic transformation *via* nucleophilic attack is kinetically hindered and would require pronounced activation of the ACE system for reducing its electron density. Likewise, oxidation may be kinetically unfavorable due to the steric hindrance of the electron-rich ring system and thermodynamically unfavorable because of its resonance stabilization. Nevertheless, considering the observed stoichiometric accumulation of SA (Figure 1C) and the detection of ANSA as an intermediate of ACE biodegradation in the isolated bacterial strains, two subsequent hydrolysis steps would be sufficient to convert ACE to easily degradable compounds. This route proceeds *via* the initial nucleophilic attack of either the sulfamate sulfur or the C〓C system (Figure 2) leading to ANSA as first hydrolysis product, which was also detected as transformation intermediate in the study by Castronovo et al. (2017). Subsequently, ANSA could be hydrolyzed to SA and the common metabolite acetoacetate. Alternatively, although less favored according to the electron density distribution in the ACE anion, hydrolases might first attack the carbonyl carbon of the amide and then the sulfamate group *via* the intermediate formation of 3-(sulfamoyloxy)crotonic acid (SOCA; Figure 2). Finally, assimilation and dissimilation of the carbon require CoA activation to acetoacetyl-CoA, which could be either reduced to 3-hydroxybutyryl-CoA or cleaved to two molecules of acetyl-CoA. Thus, complete oxidation to carbon dioxide *via* the tricarboxylic acid cycle as well as assimilation *via* the glyoxylate or ethylmalonyl-CoA pathway would be enabled.

The enzymes involved in the proposed steps might be sulfohydrolases, hydratases, or amidohydrolases. Promiscuous sulfohydrolases attacking aryl as well as alkyl sulfates *via* S-O bond cleavage can be found within the alkaline phosphatase superfamily (van Loo et al., 2018). The hydrolysis product ANSA could also be formed by the Michael addition of water to the C〓C system catalyzed by various hydratases (Resch and Hanefeld, 2015). Furthermore, diverse groups of amidohydrolases are known to hydrolyze linear and cyclic amides, albeit characterized enzymes specific for γ- or even δ-lactams are rare (Zhu and Zheng, 2018). Examples for biodegradable cyclic sulfates and amides resembling the ACE structure are 1,3-propanediol cyclic sulfate and 5-oxoproline (Figure 2). Hydrolysis of the sulfate to the corresponding diol has been demonstrated in cultures of *Rhodococcus* sp. CGMCC 4911 (He et al., 2015), and the γ-lactam can be hydrolyzed by the widespread ATP-dependent 5-oxoprolinases (Niehaus et al., 2017). This indicates that various enzymes might be present in nature potentially capable of attacking the C-N and S-O bonds of ACE. However, due to the aforementioned structural properties, ACE obviously eludes the nucleophilic attack by most hydrolases and hydratases, as degradation seems to occur only in a few bacterial genera. This suggests activation by a more specific enzyme, possibly *via* protonation of the amide nitrogen. The latter reaction would require a strongly acidic environment at the enzyme’s active site, as the pKa of ACE is about 2 to 2.4 in water and polar solvents (Popova et al., 2012). Alternatively to hydrolysis, an oxidative process is conceivable for the initial degradation step. However, our isolates degrade ACE also under nitrate-reducing conditions. This observation and the occurrence of ANSA as degradation intermediate point to hydrolysis as the more likely process, albeit we cannot yet narrow down the degradation pathway to particular enzymes and mechanisms.

ACE is a xenobiotic compound that has been in use worldwide since the 1990s. Due to its persistence in WWTPs and mobility in the aqueous phase, it became a marker of anthropogenic impact on aquatic environments. The recently observed decrease of ACE mass loads in rivers, which is probably not due to altered ACE consumption patterns, indicates increasing attenuation as a consequence of emerging biodegradation potential (Kahl et al., 2018). From the evidence of ACE-mineralizing bacteria that can use it as sole carbon source, we conclude that it took around 25 years to evolve a microbial degradation pathway for a synthetic carbon source that is bioavailable and non-toxic, thus only recalcitrant due to its xenobiotic structure. Thus, the case of ACE is an intriguing example for the “principle of microbial infallibility,” which hypothesizes “that all organic compounds could be biodegraded if only the right organism could be found, the right enzymes induced, and the prevailing environmental and nutritional conditions for its growth on that substance were suitable” (Painter, 1974) or as originally stated by Gale (1951): “It is probably not unscientific to suggest that somewhere or other some organism exists which can, under suitable conditions, oxidise any substance which is theoretically capable of being oxidised.” Based on that principle, one might argue that ACE has been biodegradable since the beginning but only the environmental conditions were not suitable before biodegradation was observed. However, biological wastewater treatment technology has not substantially changed during the period in which ACE mass loads in rivers decreased (Kahl et al., 2018). Thus, it is reasonable to assume that the emerging ACE biodegradability is a consequence of an evolutionary process. The main driver might have been the environmental conditions and the relatively high ACE concentrations in WWTPs compared to other micropollutants, as the ACE degraders seem to thrive in habitats that are already depleted in other carbon sources (Kahl et al., 2018).

Further experiments are required to identify the ACE-degrading enzymes and their genetic background, together with further efforts to isolate and characterize ACE degraders from diverse locations. To elucidate the degradation pathway, the metabolite ANSA has to be quantified, and it has to be analyzed whether SOCA or other intermediates occur as well. Once the relevant enzymes and their genes are identified, it will be possible to survey WWTPs worldwide and trace back the evolutionary origin as well as the mechanisms of global distribution and establishment of such a new catabolic trait.

## Data Availability Statement

The datasets generated for this study can be found in the GenBank acc. nos. MN270877; MN270878; MN270879; MN270880.

## Author Contributions

SK conceived the microbiological experiments, analyzed the data, and drafted the manuscript. TRo developed hypotheses on the degradation pathway and contributed to writing the manuscript. UL isolated and identified the bacterial strains and performed the biodegradation experiments. BS performed the mass spectrometry analyses and analyzed the data. TRe conceived the study, developed hypotheses on the recalcitrance and degradation mechanisms, and contributed to writing the manuscript. All authors have read and approved the final manuscript.

### Conflict of Interest

The authors declare that the research was conducted in the absence of any commercial or financial relationships that could be construed as a potential conflict of interest.
